# He-Ne Laser Seed Treatment Improves the Nutraceutical Metabolic Pool of Sunflowers and Provides Better Tolerance Against Water Deficit

**DOI:** 10.3389/fpls.2021.579429

**Published:** 2021-05-17

**Authors:** Saqib Mahmood, Beenish Afzal, Shagufta Perveen, Abdul Wahid, Muhammad Azeem, Naeem Iqbal

**Affiliations:** ^1^Department of Botany, Government College University, Faisalabad, Pakistan; ^2^Department of Botany, University of Agriculture, Faisalabad, Pakistan

**Keywords:** He-Ne seed, treatment, nutraceuticals, phenolics, sunflower, fatty Acids, water, deficit

## Abstract

Water-scarce areas are continually increasing worldwide. This factor reduces the quantity and quality of crops produced in affected areas. Physical seed treatments are considered economical and ecofriendly solutions for such problems. It was hypothesized that a moderately drought-tolerant crop grown from seeds treated with a He-Ne laser utilizes water-limited conditions better than plants grown from untreated seeds. A field study was conducted, growing a moderately drought tolerant crop (sunflower) with supportive seed treatment (He-Ne laser treatment at 300 mJ) for 0, 1, 2, and 3 min. Thirty-day-old plants were subjected to two irrigation conditions: 100% (normal) and 50% (water stress). Harvesting was done at flowering (60-day-old plants) at full maturity. The sunflowers maintained growth and yield under water limitation with a reduced achene number. At 50%, irrigation, there was a reduction in chlorophyll *a*, *a*+*b* and *a*/*b*; catalase activity; soluble sugars; and anthocyanin, alongside elevated proline. The improved chlorophyll *a*, *a*+*b* and *a*/*b*; metabolisable energy; nutritional value; and yield in the plants grown from He-Ne-laser-treated seeds support our hypothesis. Seeds with 2-min exposure to a He-Ne laser performed best regarding leaf area; leaf number; leaf biomass; chlorophyll *a*, *a*+*b* and *a*/*b*; per cent oil yield; 50-achene weight; achene weight per plant; carotenoid content; and total soluble phenolic compound content. Thereafter, the leaves from the best performing level of treatment (2 min) were subjected to high-performance-liquid-chromatography-based phenolic profiling and gas-chromatography-based fatty acid profiling of the oil yield. The He-Ne laser treatment led to the accumulation of nutraceutical phenolic compounds and improved the unsaturated-to-saturated fatty acid ratio of the oil. In conclusion, 2-min He-Ne laser seed treatment could be the best strategy to improve the yield and nutritional value of sunflowers grown in water-limited areas.

## Introduction

Recent environmental fluctuations have reduced rainfall, particularly in arid and semiarid areas, thus generating stress in plants, namely stunted plant growth, physiology, and yield. Reduced crop yield under low water availability is associated with metabolic variations in plants (Ali et al., 2020; Arslan et al., 2020). To resolve this issue, drought-tolerant crops are grown in the affected areas.

Sunflower is a moderately drought tolerant crop (Moschen et al., 2017). It is an oil-rich crop that is well-known for its nutraceutically important metabolites along with the taste and popularity of the oil. Its leaves have been used in traditional medicine due to the presence of secondary metabolites including alkaloids, tannins, steroids, and sesquiterpenoids (Ngibad, 2019). Despite being moderately drought tolerant, the negative impact of water limitation has been reported in literature. Hence, strategic solutions to improve the yield of stressed sunflowers are required.

In general, the most practiced strategies for enhanced plant production are chemical. These chemical applications can exert unpleasant effects upon the environment and the economy (Wang et al., 2020). Growing awareness of environmental ethics has motivated the move to replace chemical fertilizers with ecofriendly options. There is a modern trend to combine technology with ecological ethics by using different physical approaches. Such physical factors control and modulate the biological behavior of plants during growth and development (Cwintal et al., 2010). Keeping in mind the vital role of the light spectrum upon plant development, laser irradiation has been used as a successful pre-sowing seed treatment.

He-Ne laser seed treatment provides red light to seeds in an artificial light environment. It can manipulate plant metabolism by switching on and off certain metabolic pathways. Treated seeds show better mechanical strength (Dziwulska-Hunek et al., 2021) with positive outcomes in a number of species including sunflower (Perveen et al., 2011; Afzal et al., 2019; Hasan et al., 2020). Different combinations of red and white light variably affect growth, photosynthetic pigments (He et al., 2020), and the phytochrome-mediated physiological responses of normal and stressed plants (Możdżeń et al., 2020). However, information regarding the effect of He-Ne laser seed treatment on the levels of nutraceuticals in sunflowers subjected to drought stress has not been reported.

Primary and secondary plant metabolism responds to variations in the applied light spectrum. This response is species and dose specific (Ali et al., 2020; Danziger and Bernstein, 2021). Metabolomic profiling is one of the advanced, sensitive, and reliable technologies. It provides accurate information about possible manipulation in plant metabolism but the literature lacks information regarding the manipulation induced with the combination of He-Ne seed treatment and water limitation in sunflowers. Therefore, the study aimed to optimize the He-Ne laser exposure time for sunflower seeds, followed by the assessment of its effect upon the metabolic manipulation of normal and water-stressed sunflowers.

## Materials and Methods

### Seed Source and Selection of Irradiation Levels

The current study was conducted in the experimental field of Government College University, Faisalabad, Pakistan. Seeds of sunflower (FH-129) were obtained from Ayub Agricultural Research Institute, Faisalabad, Pakistan. Only uniform and healthy seeds were subjected to irradiation with a He-Ne laser (model no. 1508P-1256, JDS Uniphase United States) in the Department of Physics, University of Agriculture, Faisalabad, Pakistan. The seeds were exposed one by one to a continuous 300 mJ beam of light (632.8 nm wavelength and a beam diameter 1.5 mm) for 1, 2, and 3 min, following the method of Chen and Wang (2003). The experiment was designed in randomized complete blocks with four replicates. Seeds were sown and grown in two equally sized plots. With 30-day-old seedlings, one of the plots was subjected to 50% irrigation (drought stress) until maturity while the other was normally irrigated (100% irrigation). The recommended doses of fertilizer (150-60-60 NPK kg ha^–1^) were applied. At planting time, one third of nitrogen (N) as urea was mixed in the soil, while the remaining dose was split into two applications: one applied at the vegetative stage (20 days after sowing) and the second at the flowering stage. The phosphorus (P) and potassium (K) fertilizer components were applied as Triple Super Phosphate and sulfate of potash (K_2_SO_4_), respectively, at the time of seed bed preparation.

All culture conditions such as irrigation, weed management, hoeing and plant protection were kept consistent for all treatments during the experiment. The growth and biochemical attributes were studied at the flowering stage. The final harvest was collected when the back of the sunflower heads had turned yellow and the bracts were brown and dried for 4–5 days. Five capitula were selected to determine the yield of different components. After measuring the cake weight, achenes were separated. The number of achenes per capitulum was counted and the weight of 50 achenes was measured. The individual parameters were quantified by averaging five readings as technical replicates. There were three biological replicates for each data point (*n* = 3). However, for the phenolic and fatty acid profiling, due to limited quantities, the technical replicates were pooled to make a single sample and were subjected to analysis.

### Photosynthetic Pigments

The leaf chlorophyll *a* and *b* contents were determined by following Lichtenthaler (1987) and the carotenoid content was estimated with the method described by Davis (1976). Fresh leaf tissue (0.5 g) was extracted in 10 mL 80% acetone, filtered, the volume adjusted to 10 mL and the absorbance was recorded at 470, 646.8, and 663.2 nm using a spectrophotometer.

### Total Phenolic, Flavonoid, and Protein Contents

The total phenolic content of the leaves was determined according to Julkunen-Tiitto (1985) by using the Folin–Ciocalteu reagent. Acidified methanolic leaf extract (3 mL) was used. After a 90-min incubation, the absorbance was measured at 765 nm using a spectrophotometer. The total flavonoid content of leaves was determined by following the detailed procedure of Marinova et al. (2005). The total protein content of leaves was estimated with the method reported by Bradford (1976). Fresh leaves were digested in a buffer solution and the reaction mixture was prepared. The absorbance of the samples was determined at 595 nm using a spectrophotometer.

### Reducing Sugar and Total Soluble Sugar Contents

The reducing sugar content of the leaves was determined as described by Miller (1972) using 3,5-dinitrosalicylic acid (DNS). The reaction mixture was vortexed and the absorbance was measured at 540 nm using a spectrophotometer. Total soluble sugars were estimated with the method reported by Yoshida et al. (1971). After vortexing, the reaction mixture was heated at 95°C for 15 min. The absorbance was measured at 625 nm.

### Total Anthocyanin Content

The quantitative analysis of the anthocyanin content of the leaves was performed spectrophotometrically following the method described by Nozzolillo (1978). Acidified methanolic leaf extract (3 mL) was used and the absorbance was recorded at 536 and 600 nm with a spectrophotometer.

### Determination of Oil Content

The per cent oil content of leaves was determined by following the method described by AOAC (1996). Three grams of leaves were added to 30 mL *n*-hexane in a 50 mL tube. The samples were then shaken at 100 rpm for 24 h. The sample was centrifuged and the supernatant was collected. Two successive repetitions of extractions were performed using the residue by vortexing and centrifugation. A clear extract from all three collections was preserved for oil estimation. The residue was dried to determine the fat-free sample difference in weight after extraction. This value was recorded as the fat content. This procedure was repeated with a seed sample to determine the per cent oil yield.

### Nutritional Value and Metabolizable Energy

The nutritional value was determined with the method of Indrayan et al. (2005) as:

Nutritional value = (4× % age of protein) + (9× % age of fat) + (4× % age of carbohydrates).
The metabolizable energy of samples was determined with the following equation by the World Health Organization (1985).
Energy (kJ) = (4 kcal/g × g protein × 4.186) + (4 kcal/g × g fat × 4.186) + (9 kcal/g × g carbohydrate × 4.186)

### Growth and Yield

Plant growth in terms of fresh leaf weight and dry leaf weight, as well as yield attributes like capitulum weight and achene weight, were determined. The number of achenes per capitulum were counted.

### Free Proline

Free proline was determined following the method described by Bates et al. (1973). Frozen leaves (0.2 g) were homogenized in 5 mL 3% sulphosalicylic acid using a mortar and pestle. After centrifugation, the supernatant was collected. One milliliter sample, 1 mL ninhydrin, and 1 mL glacial acetic were combined and incubated in a 100°C water bath for 1 h and then shifted to an ice bath. Two milliliters of toluene were added and the mixture was incubated at room temperature for 30 min until two layers formed. The absorbance of the colored complex was measured at 520 nm. The same sequence was run with a blank (2 mL toluene). A standard curve was constructed using a proline standard. The free proline concentration was determined with the following formula:

$$\mathrm{Proline}\,\left(\mu \,\mathrm{moles}/\mathrm{gfreshweight}\right)=\frac{\mu \,\mathrm{gproline}/\mathrm{mL}\times \mathrm{mLtoluene}}{115.5\mu g/\mathrm{mole})/\mathrm{gsample}/5}$$

### Catalase (CAT) Activity

Frozen fresh leaves (0.2 g) were added to 5 mL 50 mM cooled phosphate buffer (pH 7.8). The mixture was ground using a tissue grinder mortar and pestle it was placed over an ice bath for the duration of antioxidant enzyme extraction. The mixture was centrifuged at 14000 *g* for 10 min at 4°C. The supernatant was stored in Eppendorf tubes at −20°C and was utilized to determine the CAT activity by calculating the conversion rate of hydrogen peroxide (H_2_O_2_) to water (H_2_O) and oxygen (O_2_), according to protocol by Chance and Maehly (1955). The reaction mixture was prepared by using 300 μL 30% H_2_O_2_ mixed in 200 mL phosphate buffer (pH 7.0). Subsequently, 0.1 mL of supernatant was added to 3 mL reaction solution. Then the reaction was started by the addition of enzyme extract. The absorbance at 240 nm was measured every 60 s. CAT activity is described as an absorbance change of 0.01 units per min.

$$C\,A\,T=\frac{\Delta \,A\,240\times V\,I}{W\times V\,s\,P\,0.01\times I}$$

### Phenolic Profiling

High-performance liquid chromatography (HPLC) was used for phenolic profiling. The internal standards were quercetin, gallic acid, caffeic acid, benzoic acid, vanillic acid, cinnamic acid, syringic acid, *p*-coumaric acid, *m*-coumaric acid, ferulic acid, sinapic acid, and chlorogenic acid. Phenolic compounds were extracted following the method described by Stalikas (2007) with minor modification. Powdered leaves were extracted in 100% methanol (1:10) and were filtered with a 0.45 um cellulose acetate filter (EMD Millipore, Billerica, MA, United States). An aqueous suspension of the extract was then prepared with double distilled water, the pH was adjusted to 2 with 6 M HCl and the mixture was incubated at 100°C for 3 h. All of the standards and extracts were filtered through a 0.45 μm syringe membrane filter (Millipore) and sonicated for 15 min in a Micro clean 109 bath prior to HPLC. Phenolic compounds were analyzed using gradient HPLC (LC-10AT, SCTL, Shimadzu, Japan). Elution was done for 60 min with a flow rate of 1 mL/min in a gradient system of two mobile phases: A (H_2_O_2_:Acetic acid 94:6, pH 2.27) and B (100% acetonitrile).

### Fatty Acid Profiling

Fatty acids were profiled using gas chromatography (GC), namely a Perkin Elmer gas chromatograph model Clarus 500 fitted with an Rt-2340 NB (RESTEK Corp., United States) methyl-lignocerate-coated (film thickness 0.20 μM), polar capillary column (60 m × 0.25 mm), and a flame ionization detector (FID). Oxygen-free nitrogen was used as a carrier gas at a flow rate of 67.4 psi. Other conditions were: initial oven temperature, 80°C; final temperature, 210°C; ramp rate, 3°C/min; injector temperature, 210°C; and detector temperature, 220°C. Fatty acid methyl esters (FAMEs) were determined by analyzing the relative and absolute retention times with authentic standards bought from Sigma-Aldrich (Buchs, Switzerland).

#### Preparation of FAMEs

The IUPAC (1987) protocol was followed for the preparation of FAMEs. This process involves derivatization of samples into FAMEs of triglycerides by saponification of the glycosides liberation and esterification by methanol of the fatty acids. The oil sample (0.2 g) was weighed into a 100 mL round neck round bottom flask. One pellet of KOH and 30 mL methanol were then mixed into the flask and it was refluxed for 25 min until the droplets of fat disappeared. The reaction mixture was cooled, gently shifted to a separating funnel and a small amount of *n*-hexane was added. The separating funnel was stirred gently by rotating many times and the upper layer of hexane was separated and washed three times with 10 mL deionized water. This hexane solution was dried over anhydrous sodium sulfate, filtered, and used for GC analysis. The dry and solvent-free FAMEs were preserved in a sealed sample tube in a deep freezer and used for further analysis.

### Statistical Analysis

The data were analyzed using the Costat CoHort 6.4 software. Analysis of variance (ANOVA) followed by least significant difference (LSD) was used to determine significant differences (P < 0.05).

## Results

### Growth and Yield

Statistical analysis showed all the growth parameters of untreated and treated plants growing under 100% or 50% irrigation were not significantly different (*P* > 0.05). Under 50% irrigation, the effect of He-Ne treatment was more pronounced for all growth attributes compared with 100% irrigation except for plant height, which was not different between the treatment. A 1 or 2 min He-Ne laser treatment improved the leaf area and leaf number whereas only 2-min exposure was found to be positive regarding leaf fresh weight and dry weight (Table 1).

**TABLE 1:** The growth and yield attributes of sunflowers grown from seeds subjected to pre-sowing He-Ne laser treatment.

**Stress level**	**He-Ne treatment**	**Leaf area**	**Leaf number**	**Leaf fresh weight**	**Leaf dry weight**	**Plant height**	**Capitulum weight**	**% oil yield**	**No. of achenes/capitulum**	**50 achenes weight**	**Number of achenes/capitulum**
**100% irrigation**	0 min	242.3**^*a*–*d*^**	19.0**^*abc*^**	2.00**^*d*^**	0.38**^*c*^**	100**^*ab*^**	129.3**^*bc*^**	14.8**^*b*^**	532**^*b*^**	2.55**^*a*–*d*^**	532**^*b*^**
	1 min	255.8**^*a*–*d*^**	21.0**^*a*–*c*^**	5.42**^*b*^**	1.04**^*b*^**	123**^*a*^**	024.3**^*d*^**	15.3**^*b*^**	439**^*b*^**	2.89**^*ab*^**	439**^*b*^**
	2 min	356.9**^*ab*^**	21.7**^*a*–*c*^**	8.15**^*a*^**	1.69**^*a*^**	105**^*ab*^**	028.0**^*cd*^**	14.6**^*b*^**	509**^*b*^**	2.17**^*cd*^**	509**^*b*^**
	3 min	279.1**^*bcd*^**	18.0**^*c*^**	3.62**^*bcd*^**	0.53**^*c*^**	107**^*ab*^**	051.0**^*cd*^**	15.5**^*b*^**	785**^*a*^**	2.42**^*b*–*d*^**	785**^*a*^**
**50% irrigation**	0 min	139.7**^*d*^**	14.3**^*c*^**	3.32**^*cd*^**	0.54**^*c*^**	073**^*b*^**	036.0**^*cd*^**	15.2**^*b*^**	194**^*d*^**	2.04**^*d*^**	194**^*d*^**
	1 min	317.4**^*ab*^**	26.3**^*ab*^**	3.94**^*bc*^**	0.60**^*c*^**	108**^*ab*^**	166.6**^*b*^**	17.5**^*b*^**	218**^*cd*^**	3.17**^*a*^**	218**^*cd*^**
	2 min	365.3**^*a*^**	27.3**^*a*^**	8.57**^*a*^**	1.22**^*b*^**	113**^*ab*^**	128.0b**^*c*^**	21.5**^*a*^**	330**^*cd*^**	3.00**^*a*^**	330**^*cd*^**
	3 min	271.5**^*a*–*d*^**	22.3**^*a*–*c*^**	6.06**^*bc*^**	0.82**^*c*^**	119**^*ab*^**	318.0**^*a*^**	20.5**^*ab*^**	207**^*d*^**	2.20**^*cd*^**	207**^*d*^**

*Data are presented as mean ± standard deviation (n = 3 independent biological replicates). Statistical significant differences determined by LSD (P < 0.05) are denoted by a, b, c, d.*

Like growth, almost all yield attributes were similar in both irrigation conditions for untreated plants, except for the number of achenes, which was markedly reduced with 50% irrigation. Under normal irrigation, 1, 2, and 3- min laser treatment significantly enhanced the capitulum weight, the achene weight per plant, and the number of achenes per capitulum, respectively. However, seed oil percentage and 50-achene weight did not show any response to the He-Ne laser treatment. Under 50% irrigation, there were statistically significant laser application effects. All laser treatment times enhanced the capitulum weight and the achene weight per plant. The 50-achene weight was markedly increased in plants raised from seeds exposed to 1- or 2-min He-Ne laser treatment. The 3-min He-Ne laser treatment enhanced the achene number but did not alter the 50-achene weight. The per cent oil yield was improved exclusively in plants raised from seeds exposed for 2-min to the He-Ne laser and grown under 50% irrigation (Table 1).

### Chlorophyll Contents

**FIGURE 1:**
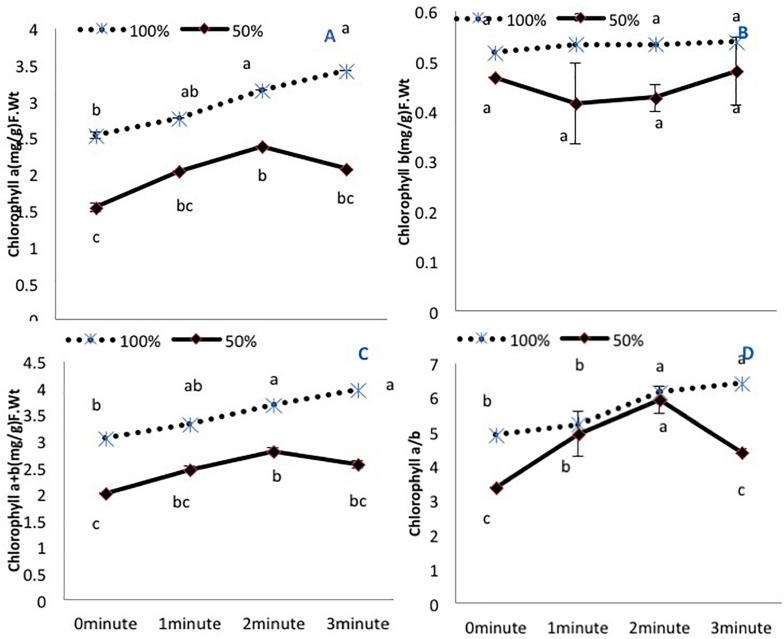
The effects of pre-sowing He-Ne laser seed treatment on pigmentation in sunflowers. For each trait, means with the same letters do not differ significantly from each other at *P* ≤ 0.05. Data are presented as mean ± standard deviation (*n* = 3 independent biological replicates). **(A)** Chlorophyll *a*, **(B)** Chlorophyll *b*, **(C)** Total chlorophyll (*a + b*), **(D)** Chlorophyll *a/b* ratio. Statistical significant differences determined by LSD (*P* < 0.05) are denoted by a, b, c.

Amongst the photosynthetic pigments, chlorophyll *a* and total chlorophyll (*a*+*b*) exhibited a decline in untreated plants grown under 50% irrigation (Figures 1A,C). A 2-min He-Ne laser exposure enhanced the content in both irrigation conditions. A 3-min He-Ne laser exposure produced positive results only when grown under 100% irrigation. The level of chlorophyll *b* was not changed based on irrigation conditions or He-Ne laser treatments (Figure 1B).

The chlorophyll *a*/*b* ratio was reduced in untreated plants grown under 50% irrigation. Under normal irrigation, 2- and 3-min He-Ne laser exposure enhanced this ratio. In water-stressed plants, only 2-min He-Ne laser treatment improved this measure (Figure 1D).

### Primary Metabolites

**FIGURE 2:**
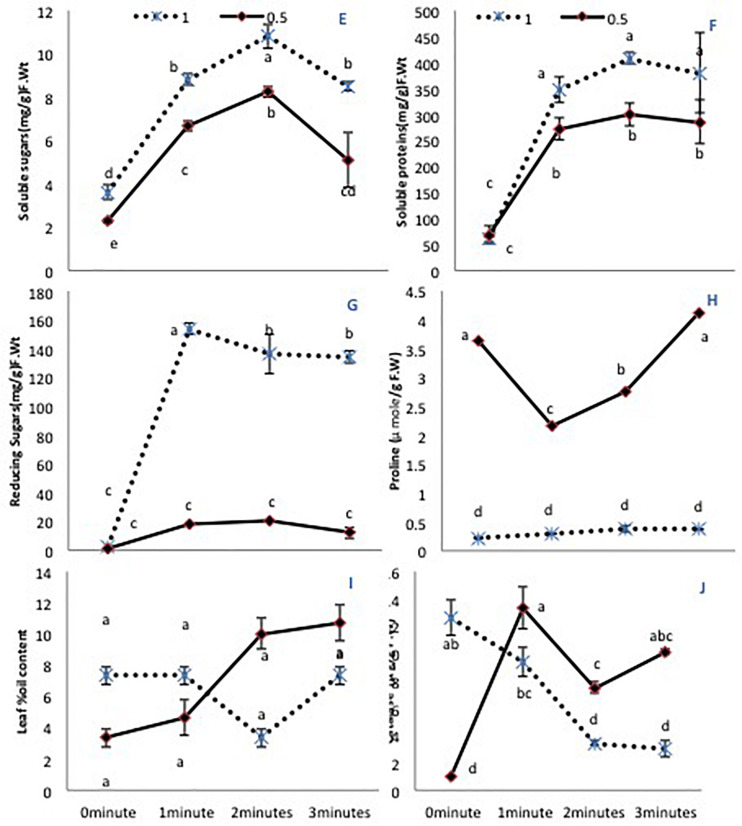
The effects of pre-sowing He-Ne laser seed treatment on primary metabolites in sunflowers. For each trait, means with the same letters do not differ significantly from each other at *P* ≤ 0.05. Data are presented as mean ± standard deviation (*n* = 3 independent biological replicates). **(E)** Soluble sugar content, **(F)** Total soluble proteins, **(G)** Reducing sugar content, **(H)** Free proline content, **(I)** Percent oil content in the leaf, **(J)** CAT activity. Statistical significant differences determined by LSD (*P* < 0.05) are denoted by a, b, c.

A limited water supply (50% irrigation) decreased significantly the total soluble sugars in untreated plants. All laser treatment times improved the soluble sugar content in both irrigation conditions (Figure 2E). Total soluble proteins showed non-significant differences when untreated seeds were grown under either irrigation condition. However, there was a significant increase (*P* < 0.01) in seeds treated with the He-Ne laser (all times) compared with untreated seeds (Figure 2F). This difference was more pronounced in plants grown under 100% irrigation compared with 50% irrigation. The reducing sugar content showed a similar increase exclusively in plants grown under 100% irrigation (Figure 2G). The free proline content showed a marked increase (*P* < 0.01) in plants grown with 50% irrigation. Under normal irrigation, none of the He-Ne laser treatments affected this measure. By contrast, in the 50% irrigation condition, 1- or 2-min He-Ne laser treatment reduced the leaf proline content (Figure 2H). In both water conditions, He-Ne laser treatment did not affect the percent oil content in the leaf (Figure 2I).

### CAT Activity

The 50% irrigation condition markedly lowered CAT activity in untreated plants. The effect of the He-Ne laser was significant for all plants but varied depending on the water supply. At 100% irrigation, He-Ne laser treatments produced a linear decline in CAT activity as the exposure time increased. At 50% irrigation, however, all laser exposures increased CAT activity (Figure 2J).

### Secondary Metabolites

**FIGURE 3:**
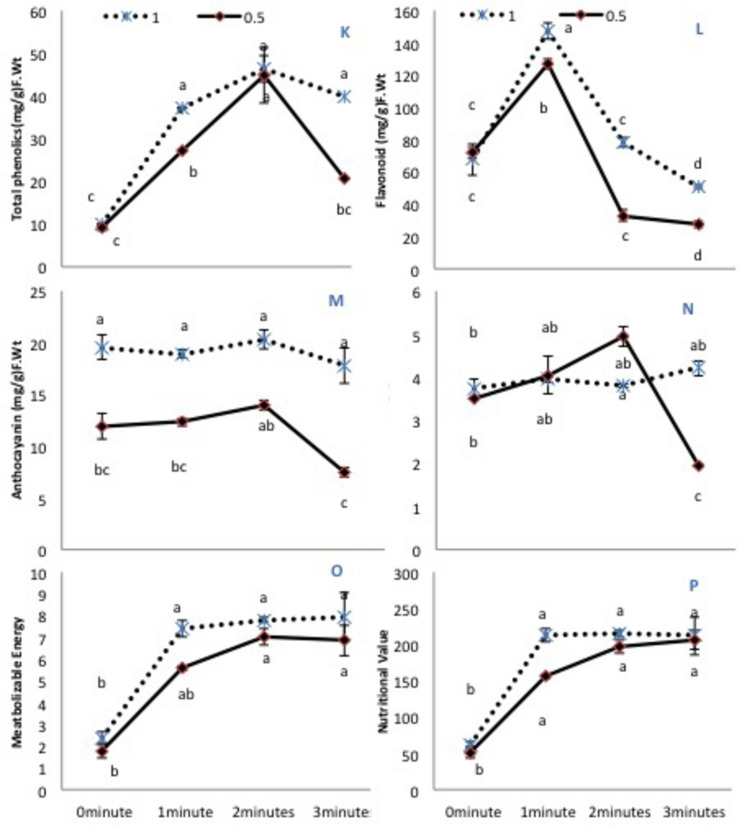
The effects of pre-sowing He-Ne laser seed treatment on secondary metabolites and nutritional value in sunflowers. For each trait, means with the same letters, do not differ significantly from each other at *P* ≤ 0.05. Data are presented mean ± standard deviation (*n* = 3 independent biological replicates). **(K)** Phenolic content, **(L)** Total flavonoid content, **(M)** Anthocyanin, **(N)** Carotenoid contents, **(O)** Metabolizable energy, **(P)** Nutritional value. Statistical significant differences determined by LSD (*P* < 0.05) are denoted by a, b, c, d.

Almost all secondary metabolites remained similar in untreated plants regardless of the irrigation conditions, except anthocyanin, which markedly declined with 50% irrigation. All He-Ne treatment times increased the phenolic content in plants grown under 100% irrigation (Figure 3K). On the contrary, in plants grown under 50% irrigation, 3-min He-Ne laser treatment produced a minimal effect, but the 1- and 2-min exposures enhanced the total phenolic content. Only 2-min He-Ne laser treatment enhanced the total flavonoid content for both irrigation conditions (Figure 3L). Overall, plants grown in 50% irrigation showed reduced anthocyanin in all treatments (Figure 3M). The carotenoid contents remained almost similar in treated and untreated plants of normally irrigated samples. However, when grown under 50% irrigation, 2-min He-Ne laser treatment enhanced the carotenoid content. This effect did not occur for 3-min exposure (Figure 3N).

### Metabolisable Energy and Nutritional Value

Metabolizable energy and nutritional value were notably increased after He-Ne treatment in both irrigation conditions. Only 1-min He-Ne laser treatment for plants grown under 50% irrigation was ineffective in increasing these measures (Figures 3O,P). Based upon the above-mentioned biochemical attributes, the phenolic and fatty acid profile of plants raised from seeds exposed to the He-Ne laser for 2 min was studied further.

### Phenolic Profile

**FIGURE 4:**
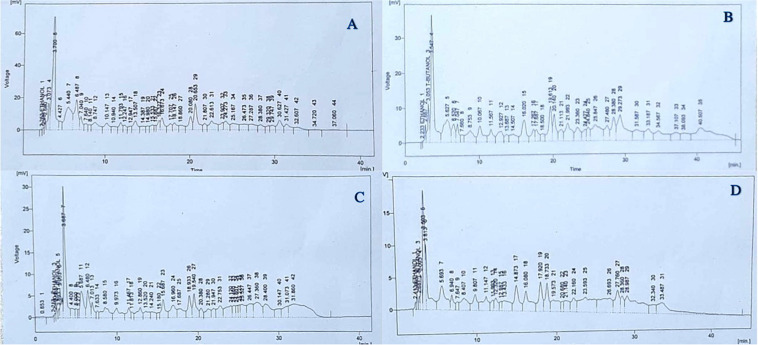
The effects of pre-sowing He-Ne laser seed treatment on the phenolic profile in sunflowers. The panels show data from untreated, 100% irrigation **(A)**, untreated, 50% irrigation, **(B)** treated, 100% irrigation **(C)** and treated, 50% irrigation **(D)** sunflowers. Data are presented as mean ± standard deviation (*n* = 3 independent biological replicates).

As shown in Figure 4B, there was a 26.6% reduction in total identified soluble phenolic compounds in untreated plants grown under 50% irrigation compared with untreated, normally irrigated plants (Figure 4A). In the case of treated plants, individual phenolic compounds responded differently to He-Ne seed laser treatments and irrigation conditions. Under 50% irrigation, untreated plants showed increases in quercetin (34.8%), gallic acid (100%), vanillic acid (62.3%), chlorogenic acid (100%), syringic acid (100%), *p*-coumaric acid (5.59%), *m*-coumaric acid (6.29%), cinnamic acid (81.2%) and sinapic acid (100%) (Figure 4B). However, there was a decline in caffeic acid (−121.1%), benzoic acid (−146.5%), and ferulic acid (−0.04%) in untreated plants grown under 50% irrigation (Figure 4B). In plants grown from He-Ne-treated seeds under 100% irrigation, there were increases in quercetin (57.6%), gallic acid (43.6%), vanillic acid (63.9%), benzoic acid (100%), *p*-coumaric acid (4.34%), *m*-coumaric acid (75.9%), ferulic acid (81.1%), and cinnamic acid (80.9%) compared with control plants (Figure 4C). In addition, there were marked decreases in caffeic acid (−34.9%), chlorogenic acid (−192.9%), and syringic acid (−244.63%). In plants grown from He-Ne-treated seeds under 50% irrigation, there was an increase in quercetin (52.65%), caffeic acid (59.94%), *m*-coumaric acid (100%), ferulic acid (72.30%), and cinnamic acid (100%) compared with untreated plants (Figure 4D). However, there were marked declines in vanillic acid (−51.3%), benzoic acid (−27.7%), and *p*-coumaric acid (−303.28%).

### Fatty Acid Profile

Untreated plants grown under 100% irrigation produced oil with palmitic acid (8.01%), stearic acid (4.11%), oleic acid (36.5%), and linoleic acid (50.76%) as the major components. The unsaturated-to-saturated fatty acid ratio was 46.96. There was a decline in this ratio in untreated plants grown under 50% irrigation (43.63). A 2-min He-Ne laser treatment affected this ratio differently depending on the irrigation condition. The plants grown under 100% irrigation displayed very similar fatty acid profiles regardless of whether the seeds had been treated with the He-Ne laser. For plants grown under 50% irrigation, He-Ne laser treatment increased unsaturated fatty acids and decreased saturated fatty acids. Ultimately, there was a marked increase in the unsaturated-to-saturated fatty acid ratio to 53.48 (Table 2).

**TABLE 2:** The fatty acid profile of sunflowers grown from seeds subjected to pre-sowing He-Ne laser treatment.

**Irrigations (%)**	**Seed treatment**	**Palmitic acid (%)**	**Stearic acid (%)**	**Oleic acid (%)**	**Linoleic acid (%)**	**Sum**	**U/SRatio**
100	Control	8.01	4.11	36.51	50.76	99.39	46.957
	He-Ne	8.02	4.13	36.56	50.26	98.97	46.956
50	Control	8.41	6.58	31.82	44.02	90.83	43.634
	He-Ne	6.96	6.16	40.69	46.19	100	53.48

*Data are presented as mean ± standard deviation (*n* = 3 independent biological replicates).*

## Discussion

In this study, the growth and metabolism of sunflowers have been compared in two irrigation conditions. Additionally, the effect of 1, 2, and 3-min He-Ne laser seed treatment were compared. A metabolomic approach was used to assess the possible manipulation manifested by both factors individually or collectively as below.

### Growth and Yield

In recent years, many crops have been raised from He-Ne-laser-treated seeds, including *Arabidopsis* (Dudareva et al., 2020), maize (Hasan et al., 2020), *Celosia argentea* (Ali et al., 2020), and sunflowers (Perveen et al., 2011). All of them have shown improved growth after He-Ne laser seed treatment, although the extent of the beneficial effects and the necessary He-Ne laser exposure time differ from species to species (Ali et al., 2020). Plant leaves are highly plastic and play a crucial role in responses to the immediate environment and stimuli. The final size and shape of treated leaves depend on their developmental pattern (Fritz et al., 2018), as observed in the current project in terms of the leaf biomass of plants grown from He-Ne-laser-treated seeds. Excess red light supplied during He-Ne laser treatment activates phytochromes, which may lead to activation of mesophyll differentiation (Carabelli et al., 2018), leaf cell expansion (Patel et al., 2013), and leaf size modulation. In addition, the activation of phytochromes may affect the leaf metabolic pathways related to DNA replication, DNA repair, cytokinesis, ribosome biogenesis, and translation (Romanowski et al., 2021). Moreover, the response of any plant toward its changing environment is based on its plasticity. This potential is further linked with phytochrome and phytohormone coordination. Plants may experience growth promotion or inhibition in such conditions. The response is mediated by a balance between growth promoters or inhibitors (Lymperopoulos et al., 2018; Ali et al., 2020). In the present work, the He-Ne laser seed treatments seemed to increase the level of growth promoters of sunflowers via phytochrome activation.

The red light (He-Ne) treatment of seeds in this study produced plants with improved leaf growth and area. These data support findings from the literature. Plants grown from He-Ne-laser-treater seeds under 50% irrigation also showed better growth and yield compared with plants grown from untreated seeds under the same water condition. These benefits may be justified in light of recent findings by Romanowski et al. (2021), who correlated activation of phyB with genes involved in DNA repair pathways during development. As observed here, the improved growth of water-stressed plants after red light treatment showed activation of phytochrome B and related DNA repair. Overall, the sunflower variety used in this experiment maintained growth and yield when subjected to 50% irrigation with only a reduction in the number of grains. These findings indicate that the variety used has moderate drought tolerance. The enhanced achene number and weight in treated plants are in agreement with Wies et al. (2019). They provided evidence of a direct correlation between light-induced phytochrome activation and leaf expansion, photosynthetic efficiency, biomass, leaf area, and grain yield.

Another supporting fact is that the role of irradiated (He-Ne) energy in cell strengthening (Dziwulska-Hunek et al., 2021) against mechanical injuries is induced by stressful conditions (Asghar et al., 2017). This laser-induced stimulation in seeds varies depending on seed type and physiology. Hence, the dose needs to be optimized for each species. In this study, 2-min exposure to the He-Ne laser seemed to strengthen the cells, denoted by enhanced oil quantity, achene weight, leaf area, leaf number, fresh leaf weight, and leaf dry weight under water stress. The higher fresh and dry weight in plants grown from He-Ne-laser-treated seeds may also be linked to enhanced photosynthetic pigments. He et al. (2020) observed enhanced chlorophyll *a*+*b* and growth rate in red and white light compared with other spectral combinations.

### Photosynthetic Pigments

Chlorophyll *a* and *b* play a central role in photosynthesis. Both are involved in light absorption (Atsushi et al., 2018). Hence, they are considered indicators of light use efficiency and production capacity (Croft et al., 2017). The reduction of photosystem II (PSII) photochemical activity is indicative of sensitivity to stress (Arslan et al., 2020). The majority of sunflower varieties maintain chlorophyll *a* and *b* levels in drought conditions. Detailed molecular studies of sunflowers have confirmed the potential of this species to delay senescence with increased expression of photosynthetic genes under stress (Moschen et al., 2017). In the present study, the cultivated sunflowers showed a reduction in chlorophyll *a*, although the level of chlorophyll *b* was not affected by water stress.

Photosynthetic pigments, particularly chlorophyll *a* and *b*, vary directly in response to spectral variations. Red and white light has been reported to produce a greater accumulation of chlorophyll *a*+*b* (He et al., 2020). In the current study, red light (He-Ne laser) treatment also increased chlorophyll *a*+*b*. In addition to the undoubted role of phytochromes in the red-light response, evidence regarding red-light-mediated stomatal responses are also available in the literature. Suppression of the red-light response was found to be associated with photosynthetic electron transport inhibition, data that support the relationship between red light and photosynthesis. It was further confirmed with variation in light intensity. The photosynthetic apparatus senses an altered redox state of its photosynthetic electron transport components like plastoquinone (PQ). PQ mediates the stomatal response in photosynthesis under red light treatment (Wang et al., 2011). Therefore, laser light treatment improves photosynthetic efficiency (Dziwulska-Hunek et al., 2021). Our findings regarding the effect of seed treatment support the literature. In a previous study in triticale, the chlorophyll *a* level was almost identical in untreated and irradiated (He-Ne) plants. Możdżeń et al. (2020) justified this outcome with the immediate transfer of excitation energy from antennae to the reaction center. In the current work, the elevated chlorophyll *a* in treated plants showed a different pattern of energy transfer in sunflower compared with triticale.

The chlorophyll *a*/*b* ratio is another indicator of plant metabolic efficiency. This ratio is positively correlated with the ratio of PSII cores to light-harvesting complex II (LHCII). PSII cores receive excitation energy from the peripheral LHCII. Hence, activation of the photosynthetic machinery may be assessed with this parameter (Souza et al., 2019). In the present study, the reduction of this ratio under stress and its improvement in He-Ne-laser-treated plants support our hypothesis. A higher chlorophyll *a*/*b* ratio indicates that better functional balance was maintained in treated plants among captured light and electron transportation as described by Terashima and Hikosaka (1995).

### Osmolyte Accumulation

Greater accumulation of osmolytes helps plants to delay senescence and upregulate a number of transcriptional elements that are involved in drought stress tolerance (Moschen et al., 2017). Leaves enriched with osmolytes like proline, soluble sugars, and proteins show better osmotic pressure and drought tolerance (Živanović et al., 2020). Therefore, the levels of these compounds are used as indexes of stress tolerance in crops.

In this study, sunflower leaves displayed a marked increase in proline under drought stress. This phenomenon indicates the possible drought tolerance of sunflowers as observed previously (Shehzad et al., 2020). A decrease in soluble sugars and maintenance of proteins in untreated water-stressed plants also pointed out moderate stress tolerance of the evaluated sunflower variety. The water stress imposition resulted in the dynamic optimization of source-sink relations (Albacete et al., 2014). Thus, the accumulation of reducing sugars was compromised and assimilated stages were used more to maintain the growth and yield under water stress conditions.

The metabolic activities of plants vary with the quality of the available light (Danziger and Bernstein, 2021). He-Ne laser treatments enhanced soluble sugar content in leaves. This outcome could be justified by the interdependence of carbohydrate metabolism and the applied irradiation (Chen, 2016; He et al., 2020). Our findings are in partial agreement with other previous reports (He et al., 2020), which describe how red light in combination with white light improved photosynthetic efficiency and carbohydrate accumulation in potato tubers.

He-Ne-laser seed treatment also enhanced soluble proteins in sunflower leaves. In a previous study, plants deficient in phytochromes (He-Ne receptors) exhibited a marked reduction in growth and total protein content (Yang et al., 2016). Those data support our finding that He-Ne laser seed treatment increased protein accumulation in sunflowers.

### Metabolisable Energy and Nutritional Value

Sunflower leaves are not a direct part of a regular diet. However, the leaves are used for herbal products and, therefore, their nutritional value may indicate safer oral use of the leaves. The nutritional value of leaves was improved with He-Ne laser seed treatment. Hence, this strategy is a safe way to improve the nutrition of sunflowers without introducing potentially toxic effects from chemical treatments. The metabolizable energy of treated plants was markedly improved, indicating a more substantial metabolic pool in the treated sunflower leaves.

### Antioxidant Metabolites

Plants cope with environmental adversities with their antioxidant metabolites in addition to their osmolytes (Prazeres and Coelho, 2020). Almost all known antioxidant metabolites under study were stable in plants subjected to drought stress, including the total phenolic, flavonoid, and carotenoid contents. These findings may be justified by the moderate drought tolerance of sunflowers, which maintained metabolizable energy under drought.

Plants grown from seeds exposed for 2 min to He-Ne laser treatment showed increased carotenoid content. This change is in accordance with Możdżeń et al. (2020). Carotenoids are antioxidants and accessory pigments of photosynthesis. Generally, in the presence of reactive oxygen species (ROS), carotenoids are converted into β-cyclocitral and other volatile and bioactive derivatives in the chloroplast. These bioactive derivatives in turn induce alterations in gene expression that alleviate stress (Ramel et al., 2012). Therefore, 2-min He-Ne laser treatment has the potential to induce drought stress tolerance in sunflowers. The key regulatory genes of carotenoid biosynthesis have been characterized in phytochromes. These structures possibly sensed the He-Ne laser light and conveyed the message of light signals to the transcriptional regulators of carotenoid synthesis (Dong et al., 2014). These regulators of carotenoid synthesis become phosphorylated leading to better carotenoid production.

Reduced CAT activity and anthocyanin content in stress also support our hypothesis that even drought-tolerant plant varieties may experience a negative effect upon growth and metabolism. He-Ne laser seed treatment improved the CAT activity of stressed plants. CAT is a known antioxidant enzyme, and an increase in its activity is directly correlated with ROS in stress and tolerance against drought-induced stresses (Prazeres and Coelho, 2020). Qiu et al. (2013) correlated this He-Ne induced activity with the overexpression of genes related to antioxidant enzymes.

### Phenolic Profile

Phenolic compounds are known secondary metabolites with pharmacological and stress tolerance potential in plants. The nutraceutical potential of the dietary phenolic compounds including ferulic acid (Kim and Park, 2019), quercetin (Tang et al., 2020), gallic acid (Kahkeshani et al., 2019), vanillic acid (Siddiqui et al., 2019), chlorogenic acid (Liang and Kitts, 2016), benzoic acid (Lima et al., 2018), *p*-coumaric acid (Pei et al., 2016), and sinapic acid (Chen, 2016) have been reported. The enhanced accumulation of these bio-molecules in He-Ne-treated plants indicated improved nutraceutical value and stress tolerance.

The secondary metabolism of plants shows sensitivity toward the quality and quantity of applied light (Danziger and Bernstein, 2021). Red, white, blue, and blue-violet light differentially affect genes, enzymes, intermediates, and products of the phenylpropanoid pathway (Taulavuori et al., 2018; Xie et al., 2020). Drought stress may also affect genes and enzymes of the phenylpropanoid pathway (Li et al., 2020). Therefore, limited water and He-Ne laser seed treatment individually and collectively showed the potential to modulate phenolic metabolism. These findings indicate that sunflower seeds can perceive sufficient energy of excitation during 2-min exposure to the He-Ne laser. This treatment modulated the phenylpropanoid pathway, manifested by altered phenolic production. A more suitable metabolic pool of He-Ne treated plants may also be justified with phytochrome B receptor-mediated activation of phytochrome B biosynthesis and improved photomorphogenesis (Gao et al., 2015).

### Fatty Acid Profile

There is an undoubtable parallel between the unsaturated-to-saturated fatty acid ratio and the nutritive value of edible oil. Molecular studies have provided evidence for He-Ne-laser-induced stimulation of morphogenesis that, in turn, participate in cell membrane repair (Pietruszewski, 2007). This repair is notable on membrane lipids too. The most observed response of fatty acids against environmental fluctuation is an alteration in the unsaturation level (Flores et al., 2007). In the present work, water limitation increased saturated fatty acids (palmitic acid and stearic acid) and reduced unsaturated fatty acids (oleic acid and linoleic acid). These outcomes are consistent with other studies that have reported the negative effect of drought stress on the unsaturated-to-saturated fatty acid ratio of other crops like *C. argentea*. He-Ne laser see treatment improved this ratio in the present sunflower samples, in accordance with previous reports Ali et al. (2020).

Oleic acid is the major unsaturated fatty acid that contributes to the nutritional value of sunflower oil. Oleic acid is synthesized by activation of D-9 desaturase and D-12 desaturase (McKeon and Stumpf, 1982). The elevated oleic acid level indicates possible activation of these enzymes after 2-min He-Ne laser seed treatment.

## Conclusion

Our findings indicate that land with limited water can be utilized to grow moderately drought-tolerant crops like sunflowers. The negative effects experienced by a stressed plant in terms of the achene number, photosynthetic pigments, enzymatic activity, and nutritive value of the oil yield may be compensated by economical treatment of seeds with He-Ne lasers (300 mJ for 2 min). In addition, this technique can be recommended due to enhancements observed in photosynthetic pigments, leaf area, leaf dry biomass, per cent oil yield, 50-achene weight, achene weight per plant, carotenoids, and total soluble phenolics, which contributed to the nutritional value of sunflower under the limited water conditions.

The limitation of the study is that it is not generalizable. The findings are species specific. Hence, for other plants the He-Ne laser dose and treatment time need to be optimized before practical implementation. In future studies, similar metabolomic experiments may be planned for structural elucidation.

## Data Availability Statement

The original contributions presented in the study are included in the article/supplementary material, further inquiries can be directed to the corresponding author.

## Author Contributions

SM and BA: sampling bench work. SP: data analysis. AW: experiment plan. NI: experimental plant and write up. MA: supervision and write up. All authors contributed to the article and approved the submitted version.

## Conflict of Interest

The authors declare that the research was conducted in the absence of any commercial or financial relationships that could be construed as a potential conflict of interest.
